# The General Infirmary, Northampton

**Published:** 1893-01-21

**Authors:** 


### The Institutional Workshop.

### WITHIN THE HOSPITALS.

### THE GENERAL INFIRMARY, NORTHAMPTON.

It not infrequently happens that much local history and some of its lore may be evolved from a study of the General Infirmary in a county town. This is true of this institution, which has been established for nearly 150 years. The library of the Northampton General Infirmary, for example, contains a most valuable and interesting collection of books, most of them old, and some of them very rare. We have often wished that we could find some member of the profession with literary tastes and leisure who would make a tour of the older county hospitals, possessing libraries, and write a series of articles, bringing out their special features of general interest, and their value to members of the profession resident in the locality. Age may be regarded in two ways. The optimist points to much good work, well done, with a considerable accumulation of funds which have been invested for the benefit of posterity. The pessimist points to the defective structural conditions and difficulties of treatment arising therefrom, the decayed buildings and the need for further extensions.

#### Structural Alterations Recommended.

The Northampton Infirmary would prove a fruitful source to both these classes of critics. It possesses too small an income, having regard to the importance and extent of the work it has to do, whilst it has more funds invested than we think altogether desirable. If our voluntary hospitals are to thrive they will continue in consequence of the support extended to them by the population in each generation as it passes. We believe that, having regard to efficiency, it is undesirable that the invested funds of any large hospital should much exceed five years' income. On this basis the Northampton General Infirmary should have £40,000 invested, and that should suffice. As it is, they have upwards of £50,000 invested, and we are sure the governors would be well advised to spend a portion of this capital at once by determining to put the older portions of the buildings into thorough repair, and at the same time to modernise the hospital and bring it up to date. This could best be done by the erecting of a new building in the centre and at the back, which should contain an accident-room and an operation theatre on the most approved principles. Some of the stone wants renewing, and the whole of the older buildings refacing, and there is urgent need for the demolition of the two small lodges, and the substitution of one good lodge with new entrance gates, &c. The time has gone by when it is justifiable to carry all the patients who have to undergo or who have undergone operations through several of the wards in sight of the occupants of the beds they contain. We do hope, therefore, that immediate steps will be taken to prepare plans for an operation theatre, and that the work will be put in hand with as little delay as possible. We are confident that such an expenditure as we have suggested would commend itself to public opinion, increase the interest already taken by the people in the infirmary, and secure a larger income than is yet obtainable both from the county and the town.

#### Recent Alterations and Additions.

Extensive alterations have been made in the Infirmary of late years, and are still being made. Thanks are due to the honorary medical staff who memorialised the governors, and pointed out to the board the bad sanitary condition of the south-east wing. Mr. C. Dorman, the architect, who has been instructed to carry out the necessary repairs and improvements, has shown considerable knowledge of modern requirements, and when the works are completed they will make these parts of the institution not only much healthier, but much better adapted to the treatment of disease. A better system of heating and a new hot-water supply has just been introduced (not before they were wanted), which must tend to increase the comforts of the patients, and to aid the work of the resident staff in many ways.

Working men, through the Hospital Week Committee, contribute about £1,100 a year to the funds, and we have no doubt that the new works, now nearly completed, will quicken their interest in the Infirmary, and that each year the sum collected in this way will increase materially. A convalescent room for the comfort of those of the in-patients who are able to leave the wards, is a special feature of this institution. It has been furnished with much care and taste by certain ladies who take an interest in the Infirmary. It is spacious, well proportioned, and an attractive feature, and should fulfil a useful purpose by aiding those patients who have arrived at a stage where disease has ceased and health is to be restored to complete a rapid recovery.

#### The Children's Ward and Out patient Department.

The children's ward, for which the senior surgeon, Dr. Percival, has worked so hard, is one of the best parts of the Infirmary. The walls and ceiling are covered with paper, and the whole has been varnished. It is therefore easy to thoroughly and frequently cleanse this ward at very small expense. Every cot, and every article of furniture contained in the children's ward, has been given, chiefly by children's organisations in the county and town. It is the practice for each Sunday-school to take charge of a particular cot, which bears a memorial brass with the name of the school upon it. Each school has collected a sum sufficient to defray the whole cost of the cot, and an annual contribution for its maintenance is received from the same source each year. It is a pleasing and creditable fact that so many schools desired to co-operate in this work that it became a little difficult to allocate the cots and assign all the articles of furniture so that everyone might provide gifts to the extent they desired. Such good works must prove beneficial to the children who take part in them, whilst they excite a widespread interest in the Infirmary, which is likely to increase and fructify exceedingly. The discipline and good order of the children's ward is remarkable, and is in striking contrast to some institutions where children are treated in considerable numbers. We congratulate the head nurse and her assistants, as the good work they do must increase the value and popularity of the whole institution. The out-patient department consists of a central hall with the usual consulting rooms and offices grouped round it. It is spacious, airy, and well-lighted, and is in every way satisfactory.

#### The Nursing Department.

Steps should be taken to erect a Nurses' Home, in connection with the Infirmary, without delay. At the present time the sleeping accommodation is altogether inadequate and unsatisfactory. The nurses have no proper sitting-room, the dining-room being a large apartment containing two large tables and so few comfortable chairs that it is practically valueless as a recreation-room. In this respect the Northampton Infirmary is far behind other county hospitals. It is not reasonable to expect really capable women to work under the present conditions if an opening should come to them to remove elsewhere. Besides, it would be most desirable for the board to determine to erect a Home, so as to enable them to have a number of probationers in training as private nurses, and thus to supply the town with an efficient staff of certificated nurses, whose capacity is known to the Infirmary authorities, as a guarantee to the local practitioners and their patients that each case of illness will be nursed by the right women in the best way. If the board should hesitate to spend a further portion of the invested capital upon the Home, then an immediate appeal should be issued to the public for funds to build it. Meanwhile, money might be raised, as in other places, by issuing 4 per cent. bonds, the interest upon which should be guaranteed by the board, the principal and interest on these bonds being made a first charge upon the profits arising from the private institution at the Infirmary. There need be no difficulty in carrying out an arrangement of this kind, which is badly wanted in Northampton, and should not only prove popular, but most useful in many ways. At any rate, under the present arrangements there is an absence of adequate sleeping and other accommodation for nurses, which ought not to be allowed to continue, or the best interests of the patients must suffer in the long run. Probationers are taken for three years' training. They receive no salary for the first year, £14 the second, and £18 the third. Pupil nurses are also taken from other institutions on payment of £5 each per quarter.

